# Topology Optimization-Based Damage Identification Using Visualized Ultrasonic Wave Propagation

**DOI:** 10.3390/ma13010033

**Published:** 2019-12-19

**Authors:** Kazuki Ryuzono, Shigeki Yashiro, Hiroto Nagai, Nobuyuki Toyama

**Affiliations:** 1 Department of Aeronautics and Astronautics, Kyushu University, 744 Motooka, Nishi-ku, Fukuoka 819-0395, Japan; Ryuzono@aero.kyushu-u.ac.jp (K.R.); nagai@aero.kyushu-u.ac.jp (H.N.); 2 National Metrology Institute of Japan, National Institute of Advanced Industrial Science and Technology (AIST), 1-1-1 Umezono, Tsukuba, Ibaraki 305-8568, Japan; toyama-n@aist.go.jp

**Keywords:** non-destructive inspection, damage identification, topology optimization, ultrasonic wave propagation, ultrasonic visualization

## Abstract

This study proposes a new damage identification method based on topology optimization, combined with visualized ultrasonic wave propagation. Although a moving diagram of traveling waves aids in damage detection, it is difficult to acquire quantitative information about the damage, for which topology optimization is suitable. In this approach, a damage parameter, varying Young’s modulus, represents the state of the damage in a finite element model. The feature of ultrasonic wave propagation (e.g., the maximum amplitude map in this study) is inversely reproduced in the model by optimizing the distribution of the damage parameters. The actual state of the damage was successfully estimated with high accuracy in numerical examples. The sensitivity of the objective function, as well as the appropriate penalization exponent for Young’s modulus, was discussed. Moreover, the proposed method was applied to experimentally measured wave propagation in an aluminum plate with an artificial crack, and the estimated damage state and the sensitivity of the objective function had the same tendency as the numerical example. These results demonstrate the feasibility of the proposed method.

## 1. Introduction

Quantitative non-destructive inspection is important to ensure the reliability and safety of structures such as aircraft and automobiles [1]. Among the non-destructive inspection techniques, ultrasonic inspection has been widely used because ultrasonic waves are highly sensitive to a damaged part and propagate over long distances. The importance of quantitative ultrasonic inspection has been described for more than 30 years [2].

To undertake quantitative evaluation by ultrasonic inspection, it is essential to develop both the measurement techniques and data analyses. In general, ultrasonic signals contain reflected waves, diffracted waves, and mode-converted waves, and some kinds of ultrasonic waves such as Lamb waves and Love waves have dispersive nature [3,4,5], which makes ultrasonic waveforms challenging to interpret. Consequently, evaluation of the damage is dependent on the skills of engineers, making misreading signals and false recognition of defects inevitable. Furthermore, the conventional ultrasonic inspection process is not automated; thus, the inspections require a lot of time and labor to scan the whole structure. Numerous studies [4,6,7,8,9,10,11,12,13] have been reported about new techniques and data analyses to overcome these difficulties. For example, ultrasonic arrays [6] have improved the inspection quality and have reduced the inspection costs by performing beam steering with a wide viewing angle through controlled transmission of multiple elements. Acoustic emission detection using a fiber-optic sensor and mode analysis [7,8,9] has also been developed to achieve a quantitative evaluation of the damage in composite laminates. The wavelet-transform has frequently been incorporated with the signal processing to analyze dispersive waves [4,10,11]. Furthermore, some inverse analyses [4,12,13] have been presented to estimate the damage quantitatively using the artificial neural network and genetic algorithm.

Aiming at further damage visibility and operability, a visualization method of ultrasonic wave propagation [14,15,16,17,18,19,20,21,22,23,24,25,26,27,28,29] has been developed. In this method, ultrasonic waves are generated by illuminating a specimen surface with a pulsed laser and are received by a fixed transducer [14]. Based on the reciprocity of wave propagation [15], the amplitude of each waveform at a particular time plotted in a contour map yields a moving diagram of the wave propagation from the receiver. The feasibility of this method was demonstrated by applying it to a crack and an artificial hollow in metallic materials [15], delamination in carbon fiber reinforced plastic (CFRP) laminates [16,17,18,19,20], a crack in welded steel plates [21], and disbonds in adhesively bonded CFRP/aluminum joints [22]. Frequency and/or wavenumber domain analysis using the Fourier- or wavelet-transform was introduced into ultrasonic propagation imaging to easily interpret the visualized results of wave propagation by isolating a specific frequency mode [23,24,25]. Moreover, a fully non-contact ultrasonic inspection [26,27,28] was demonstrated by replacing a fixed transducer with a laser Doppler vibrometer, and this method removed ringing due to the resonance of the piezoelectric transducer and made it easy to interpret scattered waves [28].

Although the visualization method of ultrasonic propagation has high damage visibility and excellent operability, it is difficult to evaluate the damage quantitatively. An efficient automatic ultrasonic image analysis has been presented using deep learning [29], but at the moment, the main target of this method is automated damage detection, not quantitative evaluation. A moving diagram of wave propagation includes wave signals at all illuminating points. Therefore, appropriate analysis for all wave signals will have the potential to acquire quantitative information about the damage.

Topology optimization [30] will be suitable for that purpose, but to our knowledge, there are few studies that apply it to damage identification. In topology optimization for structural design [31,32,33], a design domain is discretized by finite elements, and the material density distribution is assigned as design variables. Similarly, in damage identification problems, the damage severity is the design variable instead of the material density, and the damage distribution is inversely estimated by reproducing an input phenomenon of focus. Based on this idea, Lee et al. [34] demonstrated numerical examples for estimating damage in thin plates and beam models, taking resonant and anti-resonant frequencies as an objective function. Some numerical examples that focused on natural frequencies were also reported [35,36,37]. Niemann et al. [38,39,40] estimated the approximate location of the damage in CFRP laminates after impact tests. However, this damage identification focusing on frequency characteristics was not very accurate. The reason for this is the fact that frequency characteristics are not sufficiently sensitive to damage.

This study proposes a damage identification method using the visualization technique of ultrasonic wave propagation. To this end, we incorporate topology optimization with a moving diagram of wave propagation, having high sensitivity to damage. The feature of wave propagation is reproduced in the analytical model by optimizing the distribution of the damage parameters. As a result, quantitative information about the damage is estimated. This study is the first attempt to integrate the inverse analysis based on topology optimization with the ultrasonic imaging inspection. The method is first proposed, and its feasibility is then verified using a two-dimensional case of known damage location and size.

## 2. Damage Identification Procedure 

### 2.1. Concept of Damage Identification

**Figure 1:**
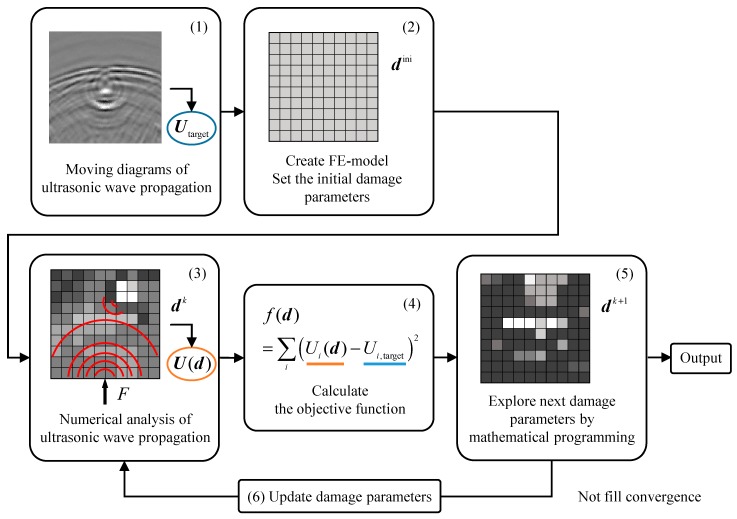
The conceptual flowchart of damage identification based on topology optimization.

Figure 1 presents the sequential process flow of the proposed damage identification method. The proposed method estimates the damage distribution that reproduces a moving diagram of ultrasonic wave propagation through the following steps. (1) Ultrasonic imaging inspection is conducted, and an ultrasonic feature in the moving diagram is adopted as target data. A potential damaged site is extracted from an extensive inspection area as the design domain. (2) An analysis model of the extracted domain is discretized into finite elements, and the initial damage parameter is assigned to each element. (3) Finite element analysis of ultrasonic propagation is performed, and the ultrasonic feature is evaluated at the present damage distribution. (4) The error between the estimated ultrasonic feature and the target data is calculated. Then, (5) the correct number of damage parameters is explored by mathematical programming. (6) The damage parameters are updated, and the process is repeated from step (3) until convergence. As a result, the position, size, and shape of damage are obtained on a per-element basis.

### 2.2. Setup of Optimization Problem

The design domain *D* is discretized into finite elements, and the damage parameter $d_{i}$ is assigned to element *i*:

(1) $$d_{i}=\{\begin{array}{c} 0\\ 1 \end{array}\;\begin{array}{c} \mathrm{for}\;i\in \Omega_{d}\\ \mathrm{for}\;i\in D\backslash \Omega_{d} \end{array}\;\begin{array}{c} \mathrm{damage}\\ \mathrm{no}\;\mathrm{damage} \end{array}$$

where $\Omega_{d}$ is the damaged domain, and $D\backslash \Omega_{d}$ is the intact domain. The solid isotropic material with penalization (SIMP) method [31] is used to relax the damage parameter, and a continuous value from 0 to 1 represents the severity of the damage. With this setting, the damage identification problem is translated into a distribution problem of the damage parameter in the design domain *D*.

The Young’s modulus of element *i* is expressed as a function of the damage parameter $d_{i}$ as:

(2) $$E_{i}(d_{i})=\left(E_{1}-E_{0}\right)d_{i}{}^{p}+E_{0},\;$$

where $E_{1}$ and $E_{0}$ are Young’s modulus of a solid (i.e., perfectly intact) and in a void (i.e., perfectly damaged), and *p* is the penalization exponent for intermediate damage. Although $E_{0}$ is initially zero, elimination of an element (i.e., modification of the model) is cumbersome in the optimization process; therefore, a small stiffness ($E_{0}=0.001\;\mathrm{MPa}$) is assigned to perfectly damaged elements.

The topology optimization problem is defined as the minimization of the square error between the estimated ultrasonic feature and the target data as follows:

(3) $$\underset{d}{\min}f(d)=\sum_{j=1}^{m}{\left(U_{j}(d)-U_{j,\mathrm{target}}\right)}^{2}\;\mathrm{subject}\;\mathrm{to}\;0\leq d_{i}\leq 1\;\mathrm{for}\;i=1,\cdots ,n$$

where $U_{j}(d)$ is the analytically obtained ultrasonic feature at node *j* with the present damage state ***d***, $U_{j,\mathrm{target}}$ is the ultrasonic feature of target data at an illuminating point, and *m* and *n* are the total number of nodes and elements in the design domain *D*. The ultrasonic feature in the objective function should be selected appropriately, depending on the problem. Although a few studies [38,39,40] used the summation of damage parameters as a constraint condition, no constraint conditions other than Equation (3) provided better results in the preliminary investigation.

## 3. Numerical Examples

To verify the feasibility of the proposed method, this section applies it to numerical examples with known damage. The target data ultrasonic feature $U_{j,\mathrm{target}}$ in Equation (3) is obtained by forward analysis of ultrasonic wave propagation in a target model. To simplify the problem, a penetrating crack in a metal plate is considered here, and all the analyses are two-dimensional.

### 3.1. Numerical Models

**Figure 2:**
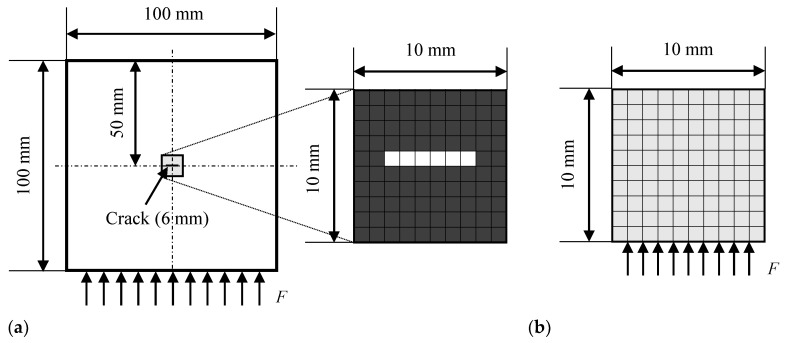
Models to calculate data of ultrasonic waves: (**a**) Target model; (**b**) Inverse analysis model.

Figure 2a shows the analysis model. The target plate model was 100 × 100 mm, and a penetrating crack 6 mm long and 1 mm wide was introduced at the center. Four-node quadrilateral solid (plane-stress) elements were used, and the size of each element was 1 × 1 mm. There were 10,000 elements and 10,201 nodes. The crack was expressed as damaged elements with a small Young’s modulus $E_{0}$. Five period-sinusoidal wave loads multiplied by the Hanning window function were applied to all nodes on the lower surface; their amplitude and frequency were 1 N and 1 MHz. A non-reflective boundary condition [41] that removes one reflection was set on the upper, left, and right surfaces. The material was assumed to be stainless steel, and its Young’s modulus, Poisson’s ratio, and density were 186.6 GPa, 0.306, and 7.86 g/cm^3^, respectively.

**Figure 3:**
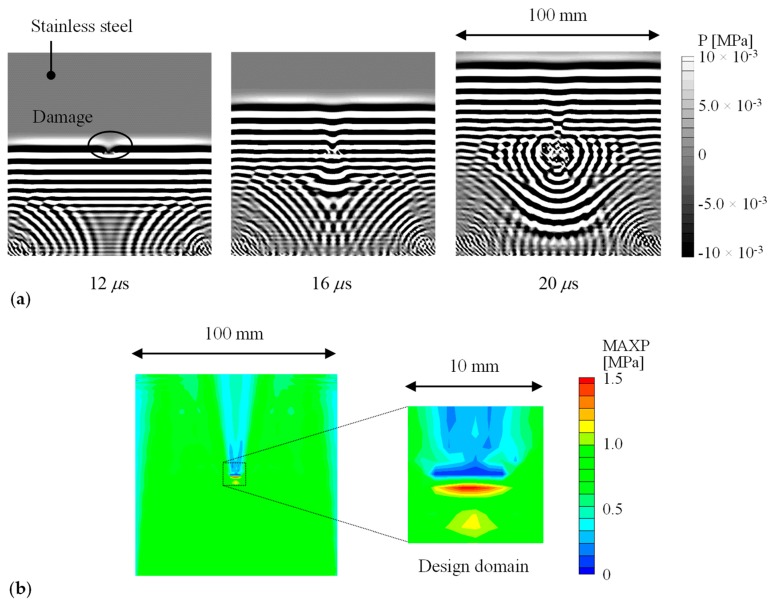
Numerical results of wave propagation on a stainless-steel plate with a crack: (**a**) Snapshots (mean stress); (**b**) Distribution of the maximum amplitude of the mean stress.

Figure 3 depicts the analyzed ultrasonic wave propagation. The ultrasounds propagated from the bottom to the top and were diffracted and scattered at the crack. The waves propagating from the bottom corners were reflected waves. Figure 3b shows the distribution of the maximum amplitude of the mean stress. The maximum amplitude had a characteristic distribution near the crack (e.g., high in the lower area and low in the upper area to the actual crack). Therefore, the crack will be reproduced by focusing on the maximum amplitude distribution.

The inverse analysis model (i.e., design domain *D*) was a 10 × 10 mm domain (including 100 elements and 121 nodes), which was extracted from the center of the target model, as illustrated in Figure 2b. Topology optimization defines the same number of design variables as elements in the design domain. Therefore, the size of the inverse analysis model and the calculation cost are in a trade-off relationship. Input loads, similar to those in the forward analysis, were applied to the lower surface of the inverse analysis model. A non-reflective boundary condition [41] was set on the upper, left, and right surfaces.

### 3.2. Damage Identification for Numerical Results

The maximum amplitude of the mean stress was adopted as the ultrasonic feature in the objective function. Thus, the optimization problem is defined as follows:

(4) $$\underset{d}{\min}f(d)=\sum_{j=1}^{m}{\left(\sigma_{j,\max}(d)-\sigma_{j,\max,\mathrm{target}}\right)}^{2}\;\mathrm{subject}\;\mathrm{to}\;0\leq d_{i}\leq 1\;\mathrm{for}\;i=1,\cdots ,n$$

where $\sigma_{j,\max}(d)$ is the maximum amplitude of the mean stress estimated at node *j* with the present damage state ***d***, and $\sigma_{j,\max,\mathrm{target}}$ is that of the target data at node *j* obtained by forward analysis (Figure 3b right) in this section. A constant value of the damage parameter, $d^{\mathrm{ini}}$, was initially assigned to all the elements, and ten inverse analyses were performed using $d^{\mathrm{ini}}$ from 0.1 to 1.0, with an increment of 0.1. The penalization exponent *p* of 3 was used as in previous studies [31,34,35,36,38]. Sequential quadratic programming in the MATLAB Optimization Toolbox (R2019a, MathWorks, Inc, Natick, MA, USA) was used for exploration and updating of the damage parameters. The gradient of the objective function was calculated by the finite-difference method.

**Figure 4:**
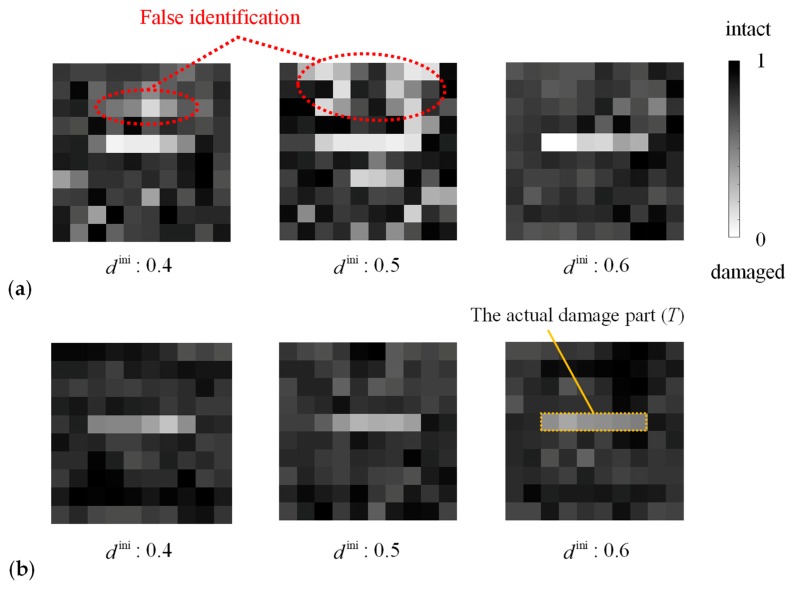
The optimal solution of the damage state obtained using ultrasonic wave data (**a**) from a single direction and (**b**) from two directions.

Figure 4a depicts typical damage distributions estimated by the proposed method. The estimated damage parameters of the elements in the actual damaged domain (*T*) were smaller than that of most elements in the actual intact domain (D\T) in all of the ten cases of the initial damage parameter $d^{\mathrm{ini}}$ distribution. This result suggests that the damage in the target can be estimated by the proposed method; however, the estimated damage states were different depending on the initial damage parameter $d^{\mathrm{ini}}$ in the upper area of D\T. Moreover, for example, in the case of $d^{\mathrm{ini}}=0.5$, the damage parameters smaller than its maximum in *T* (0.2016) were estimated in nine elements in D\T, which were erroneously determined to be damaged. Such elements were concentrated in the upper region of *D*.

**Figure 5:**
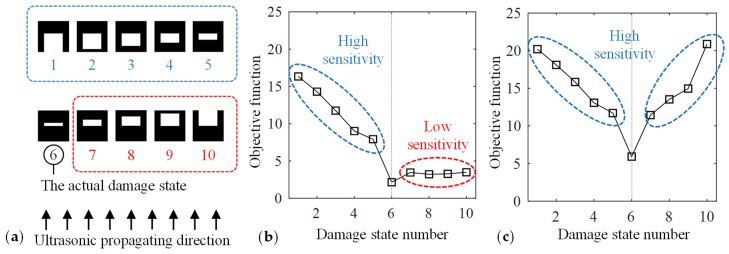
The objective function values obtained using ultrasonic wave data in (**a**) the various damage states (**b**) from a single direction and (**c**) from two directions.

The reason for this false identification is caused by the low sensitivity of the objective function in the area above the crack. The objective function was evaluated at various damage states depicted in Figure 5a. Damaged elements were added to the lower area of D\T in #1–#5 and to the upper area in #7–#10. Figure 5b presents the value of the objective function obtained by forward analysis in these damage states. The gradient of the objective function within #6–#10 was smaller than that within #1–#6. Moreover, the maximum amplitude in the area above the actual crack was smaller than that in the other areas, as shown in Figure 3b, because the diffracted ultrasonic waves propagated with a small amplitude above the crack as a result of the high directivity of ultrasound. Therefore, the maximum amplitude hardly changed in the area above the actual crack even if the damage state in that area was altered. Consequently, the damage identification results depended on the relationship between the crack geometry and the direction of ultrasonic wave propagation, and it was difficult to estimate the right damage state in the area above the crack with the objective function (4).

To improve the sensitivity of the objective function, the wave propagation from the top to bottom (superscript: upper) was considered in addition to that from the bottom to the top (superscript: lower); thus the optimization problem is re-defined as follows:

(5) $$\begin{array}{c} \underset{d}{\min}f(d)=\sum_{j=1}^{m}{\left(\sigma_{j,\max}^{\mathrm{lower}}(d)-\sigma_{j,\max,\mathrm{target}}^{\mathrm{lower}}\right)}^{2}+\sum_{j=1}^{m}{\left(\sigma_{j,\max}^{\mathrm{upper}}(d)-\sigma_{j,\max,\mathrm{target}}^{\mathrm{upper}}\right)}^{2}\\ \mathrm{subject}\;\mathrm{to}\;0\leq d_{i}\leq 1\;\mathrm{for}\;i=1,\cdots ,n \end{array}$$

In ultrasonic visualization experiments, ultrasonic propagation data from two directions can easily be recorded.

Figure 4b depicts the estimated damage states based on Equation (5). The damage parameter in D\T was always greater than its maximum in *T*, and thus, the actual damage *T* was successfully identified. However, the difference of the damage parameter in *T* and in D\T was small, and this issue will be discussed in the following section. The sensitivity of the objective function was high in both the upper and lower areas of the target crack, as shown in Figure 5c. Thus, when the sensitivity was enhanced in the entire design domain, the proposed method provided the damage identification results closer to the target damage state.

The above results and discussion demonstrate the feasibility of the proposed method. This study used the maximum amplitude distribution as the ultrasonic feature, and two sets of ultrasonic propagation data were required in the objective function to enhance its sensitivity. However, considering the cost of inspection, it is recommended to estimate damage using a single set of wave propagation data. To that end, the objective function should be improved and will be investigated in our future study.

### 3.3. Effect of the Penalization Exponent

The penalization exponent *p* in Equation (2) has been discussed in previous studies, because the relaxation of the design variables yields an intermediate density called a gray-scale element, which cannot be interpreted physically in structural design problems. To clarify the physical meaning of the gray-scale element, Bendsøe et al. [42] discussed the range of *p* based on Hashin-Shtrikman (HS) bounds [43] and proved that the SIMP method is physically permissible as long as *p* is greater than a certain value (e.g., *p* ≥ 3 for two-dimensional problems with Poisson’s ratio of 1/3). In previous studies of damage identification based on topology optimization, Nishizu and Neumann et al. [35,38,39,40] set *p* = 3, Reumers et al. [36] set *p* = 1, and Eslami et al. [37] changed *p* gradually from 3 to 1. An element (i.e., microscopic area) will have various Young’s modulus depending on the severity of the damage. Therefore, unlike structural design problems, the intermediate damage parameters are acceptable in the damage identification problem. Instead, it is essential to set a threshold to distinguish a damaged from an intact region.

**Figure 6:**
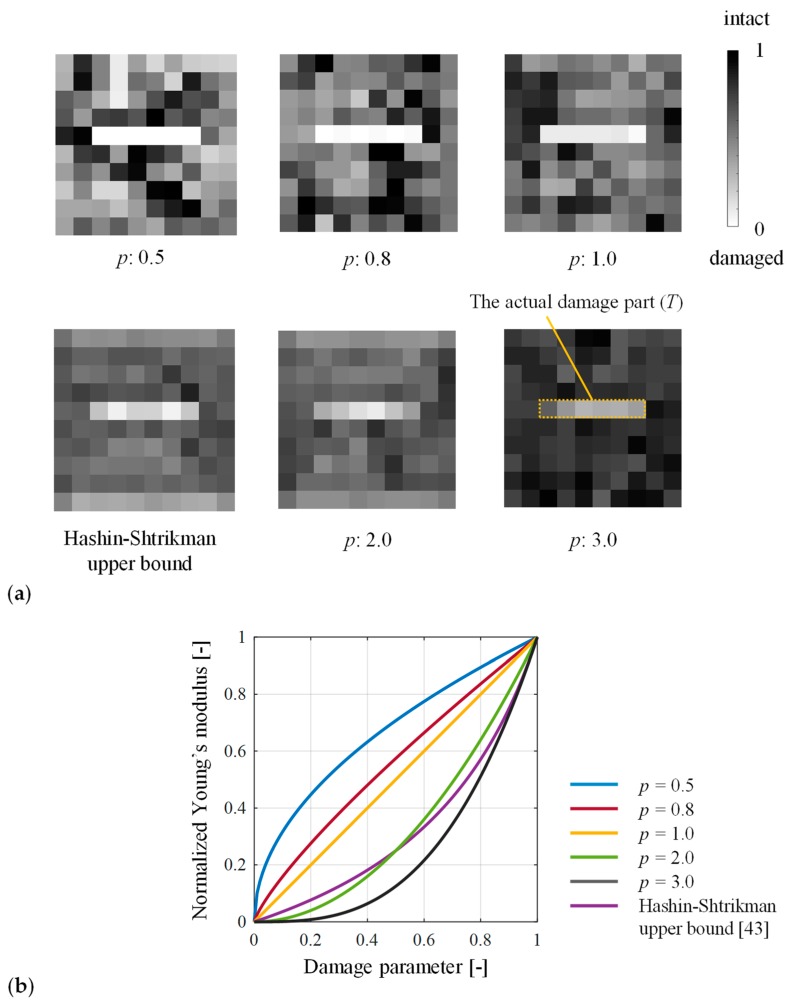
Comparison of (**a**) the optimal solution of the damage state and (**b**) the values of Young’s modulus as a function of damage parameters with the various penalization exponents and the Hashin-Shtrikman upper bound [43].

Damage identification using various penalty exponents and the HS upper bound was performed. Here, the problem is defined by Equation (5) and Figure 2; the initial value of the damage parameter, $d^{\mathrm{ini}}$, was 0.5 in all the cases. Figure 6a depicts the estimated damage states, and Figure 6b shows a variation of Young’s modulus normalized by $E_{1}$. The inverse analysis did not converge when *p* was less than 0.5 or greater than 3. In all converged results, the maximum value of the damage parameter in *T* was smaller than its minimum in D\T, and the damage state was estimated appropriately.

When *p* was greater than 1 (including the HS upper bound), the damage parameters were almost uniform in D\T. Furthermore, as *p* increased, the damage parameters in D\T approached 1, and the number of gray-scale elements decreased. This is because the gradient of Young’s modulus in damage parameters close to 1 is large when *p* = 2 or more, as shown in Figure 6b. Therefore, *p* should be greater than 2 to interpret the elements as undamaged physically.

On the other hand, when *p* was 1 or less, the difference of the damage parameter in *T* and in D\T was larger than that with *p* greater than 1. Furthermore, the damage parameter in *T* became almost 0. As shown in Figure 6b, when the damage parameter is close to 0, Young’s modulus changes significantly with a small variation in the damage parameter. Therefore, the damage parameter tends towards 0 in *T*, and it hardly approaches 0 in D\T. The penalization exponent of 0.5–1 enables setting a threshold to distinguish between damaged elements from intact ones. For example, when using *p* = 0.8 and the threshold $d_{\mathrm{th}}=0.1$, the damaged and undamaged elements were distinguished in all initial values of the damage parameter. The above discussion indicates that the penalization exponents *p* of 0.5–1 are suitable for damage identification.

## 4. Application

In this section, the feasibility of the proposed method is verified by applying it to the measured wave propagation in an aluminum plate with an artificially generated crack whose position, length, and width are known. Therefore, the experimentally measured maximum amplitude was used as the target data $\sigma_{j,\max,\mathrm{target}}$ in Equation (4).

### 4.1. Experiment

**Figure 7:**
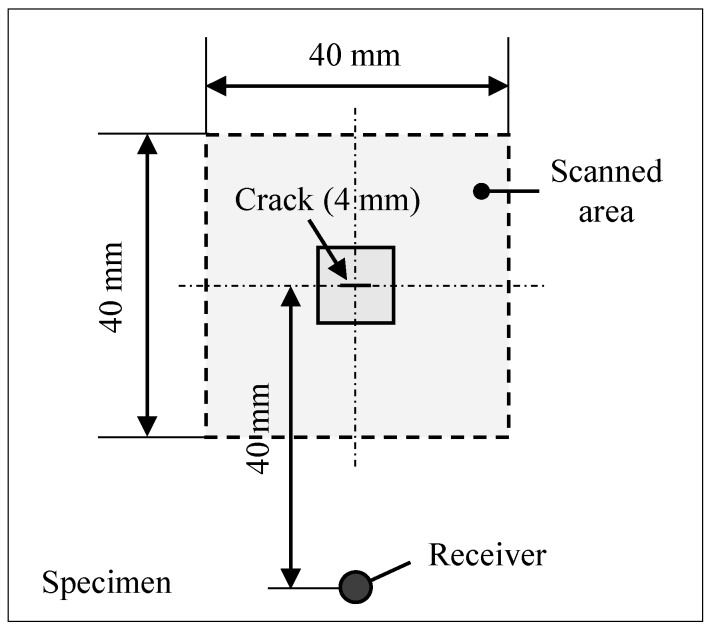
Schematic of an aluminum plate with a crack and the pulsed laser scanning area.

Figure 7 presents a schematic of the test configuration. The test piece was an aluminum plate with dimensions of 500 × 500 × 5 mm. A crack 4 mm long and less than 0.3 mm wide was introduced at the center of it. A 40 × 40 mm region, including the crack, was scanned by an Nd:YAG pulsed laser (DIVA II, Thales Laser Co., Ltd) at a constant illumination spacing of 0.25 mm (160 × 160 points). The pulse duration was 10 ns with 5 mJ energy per pulse, and the diameter of the beam spot was 2 mm. The ultrasonic wave generated by each illumination was received by a transducer placed 40 mm apart from the crack. Its resonance frequency was 5 MHz, and a 70° wedge was used. The received signals were recorded in a computer through an amplifier and a digital oscilloscope.

**Figure 8:**
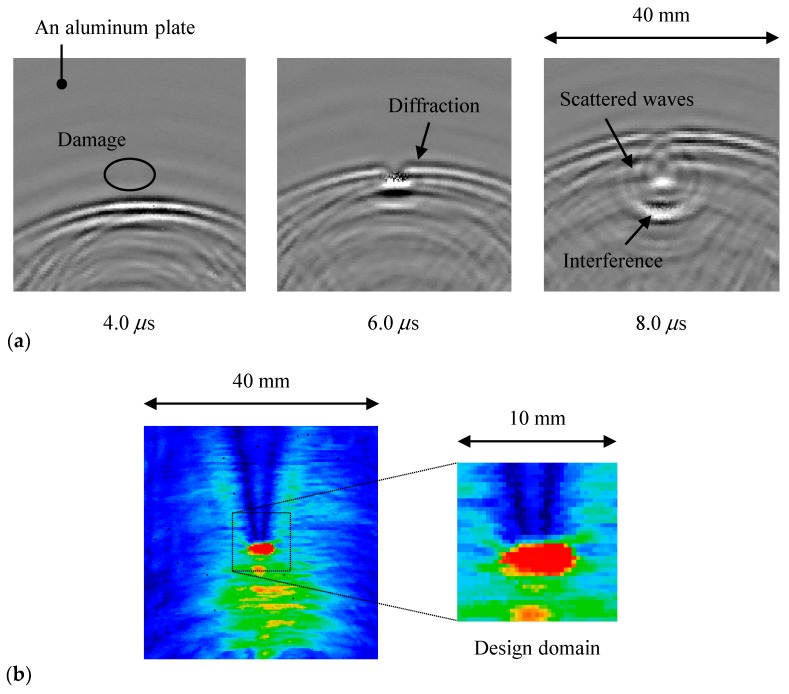
Inspection results by the visualization method of wave propagation on an aluminum plate with a crack: (**a**) Visualized results of ultrasonic waves; (**b**) Distribution of the maximum amplitude.

Figure 8a depicts the visualized results of the ultrasonic wave propagation in an aluminum plate with a crack. The traveling waves were reflected at the crack, and the amplitude below the crack was high due to the interference of the traveling and reflected waves, as shown in the maximum amplitude map (Figure 8b). In contrast, the diffracted waves propagated with a low amplitude because of the high directivity of ultrasound, hence the maximum amplitude above the crack was lesser than that in other regions. The approximate location of damage was found from the sudden amplitude change; however, it was difficult to quantitatively evaluate the length because the maximum amplitude distribution was rounded off near the crack tips, as shown in Figure 8b.

### 4.2. Damage Identification for Experimental Results

**Figure 9:**
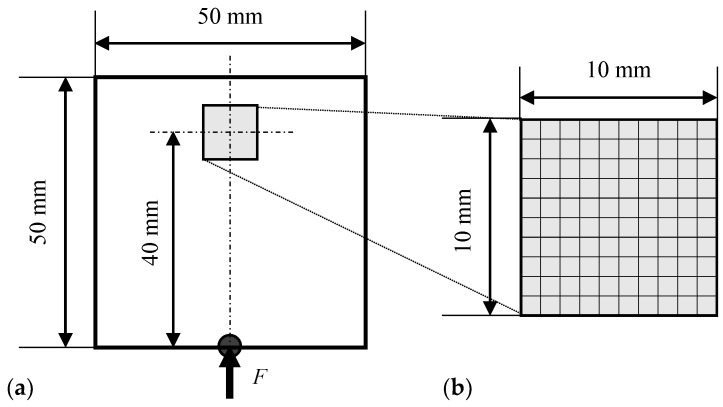
Numerical models with the same configuration as the experiment: (**a**) Ultrasonic wave propagation analysis model; (**b**) Inverse analysis model.

Figure 9a shows the two-dimensional analysis model. The dimension of the analysis domain was 50 mm long and 50 mm wide. Four-node quadrilateral solid (plane-stress) elements were used, and the size of each element was 1 × 1 mm. There were 2500 elements and 2601 nodes. Five period-sinusoidal wave loads multiplied by the Hanning window function were applied to the center point of the lower surface (i.e., the position of the receiver); their amplitude and frequency were 1 N and 5 MHz. A non-reflective boundary condition [41] that removes one reflection was set on the upper, left, and right surfaces. Young’s modulus, Poisson’s ratio, and density of the aluminum plate were 69.0 GPa, 0.34, and 2.70 g/cm^3^.

The design domain *D* was a part of the analytical model located 40 mm from the bottom, as shown in Figure 9b. Its size was 10 × 10 mm, including 100 elements. The damage parameters only in the design domain *D* were updated, and those in the other areas remained constant at unity. The size of the element was 1 mm square, and therefore, the location and length of the crack could be identified, but the width could not be estimated. Equation (4) was used as the optimization problem setting, and the maximum amplitude distribution on the right of Figure 8b was used as the target data. The initial value of the damage parameter $d^{\mathrm{ini}}$ was 0.5. The penalization exponent *p* = 0.8 was adopted based on the discussion in Section 3.3.

**Figure 10:**
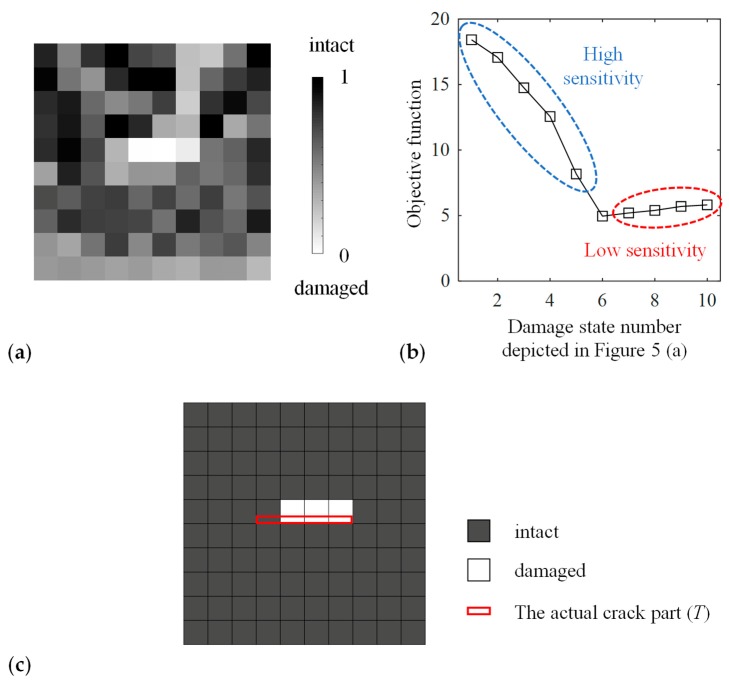
Results of applying the proposed method to experimentally measured wave propagation: (**a**) The optimal solution of the damage state; (**b**) The objective function values in the various damage states as with Figure 5; (**c**) The estimated damage state when using the threshold $d_{\mathrm{th}}=0.1$.

Figure 10a depicts the estimated damage state. The maximum value of the damage parameter in *T* was 0.2950, and there were five elements in D\T that had a damage parameter smaller than that value. Most of these elements were in the area above the actual crack, and this pattern was similar to Figure 4a. The sensitivity of the objective function above the damage was evaluated in Figure 10b in the same manner as in Figure 5. The curve had the same tendency as in Figure 5b, and the low sensitivity in the area above the actual crack caused the misestimation. Nonetheless, as shown in Figure 10c, when using the threshold $d_{\mathrm{th}}=0.1$ for distinguishing between the damaged from the intact region, three elements out of the actual damage region *T* (four elements) were estimated as damaged, and all the other elements were estimated as intact.

The feasibility of the proposed method was thus demonstrated, though these results were obtained in simple and ideal model cases. A more detailed investigation will be required for the application of the present method to real problems.

## 5. Conclusions

This study proposed a damage identification method of incorporating topology optimization with the visualization of ultrasonic wave propagation. The distribution of the damage parameter in a design domain was estimated by reproducing the ultrasonic maximum amplitude map of the target data. The proposed method was verified by applying it to numerical and experimental examples with known damage. The conclusions are summarized below:

1. The actual damage state was successfully estimated in the numerical examples. The damage was identified with high accuracy using ultrasonic propagation data from two directions to increase the sensitivity of the objective function in the whole design domain.
2. The effect of the penalization exponent *p* for determining Young’s modulus and the efficiency of the damage identification was investigated. The standard value (*p* = 3) in the structural design problems resulted in moderate damage severity in the actual damaged area. In contrast, a penalization exponent less than unity resulted in higher damage severity in that area, and the difference between the damage parameter value between ‘damaged’ and ‘intact’ regions was greater than that in the case of *p* = 3. The penalization exponent within the range 0.5 ≤ *p* ≤ 1 enabled setting a threshold to distinguish between damaged elements from intact ones.
3. The proposed method was applied to experimentally measured wave propagation in an aluminum plate with an artificial crack. The actual damaged area was estimated, and the estimated damage state and the sensitivity of the objective function had the same tendency as a similar numerical example. This demonstrated the feasibility of the proposed method.

Damage identification in simple and ideal model cases was demonstrated as the first attempt. In our future research, potential of the proposed method will be explored in general cases such as oblique incident to an embedded crack.
